# Intracardiac Echocardiography as a Guide for Transcatheter Closure of Patent Ductus Arteriosus

**DOI:** 10.1155/2020/5147193

**Published:** 2020-07-30

**Authors:** Hironaga Yoshimoto, Maeda Yasuto, Tadashi Inoue, Yoshiyuki Kagiyama, Yozo Teramachi, Ryuta Takase, Yusuke Koteda, Yoshihiro Fukumoto, Motofumi Iemura, Kenji Suda

**Affiliations:** ^1^Department of Pediatrics and Child Health, Kurume University School of Medicine, Kurume, Japan; ^2^Department of Cardiovascular Medicine, Kurume University School of Medicine, Kurume, Japan; ^3^Cardiovascular Research Institute, Kurume University School of Medicine, Kurume, Japan; ^4^Department of Pediatric Cardiology, St. Mary's Hospital, Kurume, Japan

## Abstract

**Background:**

Transcatheter closure of patent ductus arteriosus (TC-PDA), conventionally guided by aortography, has become the standard treatment of this disease. The purposes of this study were to evaluate whether intracardiac echocardiography (ICE) may be used for measuring PDA size and be used as a guide for TC-PDA.

**Methods:**

This study had 2 phases. In phase 1, we compared the measurements of PDA size: pulmonary artery side diameter (PA-D), length, and aortic side diameter (Ao-D) of PDA, as measured by ICE with those measured by aortography or cardiac computed tomography (AoG/CCT) in 23 patients who underwent TC-PDA. In phase 2, we compared the demographics, fluoroscopic time, contrast volume, and complications of the TC-PDAs between 10 adult patients with ICE guidance and 16 without it.

**Results:**

In phase 1, we found great correlation and agreement between ICE and AoG/CCT in PA-D (*r* = 0.985, bias −0.077 to 0.224), but moderate to poor correlation and agreement in length (*r* = 0.653, bias −0.491 to 3.065) and Ao-D (*r* = 0.704, bias 0.738 to 4.732), respectively. Nevertheless, all patients underwent successful TC-PDA with ICE guidance that allowed us to continuously monitor the whole process. In phase 2, TC-PDA required a significantly lower contrast volume with ICE guidance than without it, and there was no significant difference in the remaining variables between the 2 groups.

**Conclusion:**

ICE is comparable to AoG/CCT in providing accurate PA-D of the PDA and may be a safe alternative to guide TC-PDA as compared to conventional aortography.

## 1. Introduction

Patent ductus arteriosus (PDA) is the persistence of fetal circulation between the systemic and pulmonary circulations. Since Porstmann et al. performed the first transcatheter closure of PDA (TC-PDA) without a thoracotomy in 1967 [1], it has become a widespread procedure and is currently the standard treatment [2–6]. Accurate PDA size measurements are very important in selecting devices for TC-PDA [6–8].

However, in adults with large PDAs, aortography requires a significant amount of contrast and yet may not provide an adequate image for evaluating PDA anatomy [8, 9]. This may lead to underestimations of PDA size and resultant embolization or exchange of the device [9]. In addition, aortography is precluded in patients with anaphylaxis or renal dysfunction [5, 10, 11].

Glassman and Kronzon first implemented intracardiac echocardiography (ICE) with a microtransducer mounted on a wire to guide transseptal punctures in a cardiac catheterization laboratory [12]. An advantage of ICE is its imaging from within the heart; it also provides shorter image distances and higher resolutions. Also, ICE can provide detailed cardiovascular anatomy images without general anesthesia and tracheal intubation. ICE is now commonly used to guide transcatheter closures of atrial septal defects and transseptal punctures for radiofrequency ablations [13–15].

Recently, we developed novel techniques for using ICE as a guide for various types of catheter interventions such as TC-PDA and stent placements into the superior vena cava as well as in coarctation of the aorta [16–18]. We have proposed novel imaging views using ICE in TC-PDA, including the main pulmonary artery view (MPA view) which is equivalent to short axis view in transthoracic echocardiography and the left pulmonary artery view (LPA view) which is equivalent to long axis view in a small series of patients [16]. However, no studies have compared ICE with aortography or cardiac X-ray computed tomography (CCT) to evaluate PDA anatomy and be a guide for TC-PDA.

Therefore, this study compared ICE with aortography/CCT for measuring PDA sizes and evaluated the efficacy and safety of ICE-guided TC-PDAs.

## 2. Methods

### 2.1. Patient Selection

There were 2 phases in this study. In phase 1, we sequentially enrolled 23 patients (13 children and 10 adults) who weighed more than 15 kg and underwent TC-PDA at the Kurume University and St Mary's Hospitals between April 2014 and April 2019. Patient demographics are described in Table 1. In phase 2, we enrolled all 10 adult patients in phase 1 and sequential 16 adult patients who underwent TC-PDA without ICE guidance between 2009 and 2013 at our hospitals. This study was approved by the institutional review boards of both hospitals. Written informed consent was obtained from all patients or their parents, where applicable.

### 2.2. Cardiac Catheterization Procedures

All procedures were performed under local anesthesia. The subjects were administered with intravenous 50 UI/kg heparin and antibiotics before the procedure. The right femoral artery and vein were cannulated with 4 and 6 French (Fr) sheaths, respectively.

In patients who underwent CCT and had sufficient information of PDA anatomy before TC-PDAs, we proceeded to right heart catheterization, but in patients without CCT before TC-PDA and in some patients with CCT who were thought to have insufficient information, a 4 Fr pigtail catheter was advanced retrogradely, and a descending aortography was performed in 30° right anterior oblique and straight lateral views at 30 frames per second to measure PDA sizes before the right heart catheterization.

Then right heart catheterization was performed with an end-hole catheter (Arrow International, Inc., Reading, PA, USA) under fluoroscopic guidance. A hemodynamic study and a complete oximetry run were performed, and the cardiac output and pulmonary to systemic blood flow ratios were determined using the Fick principle. Following this, we proceeded to TC-PDA under ICE guidance.

### 2.3. ICE Imaging

In patients weighing more than 20 kg, another 8 Fr short sheath or 9 Fr long sheath was placed in the same femoral vein; however, in the remaining patients, an 8 Fr short sheath was placed in the other femoral vein under the ultrasound guidance.

The detailed procedure for using ICE to evaluate PDAs has been discussed in a previous report [16]. When we used 8Fr short sheath, an ICE catheter was inserted through the given sheath and advanced into the heart. We started from a home view, which depicted the right heart from the right atrium [14, 15]. After the home view was obtained, we advanced the ICE catheter further into the MPA to obtain the MPA views [16]. When we used 9 Fr long sheath, the tip of the sheath was placed into the MPA using a set of balloon catheter and guidewire, through that we advanced ICE catheter and obtained MPA view.

**Figure 1:**
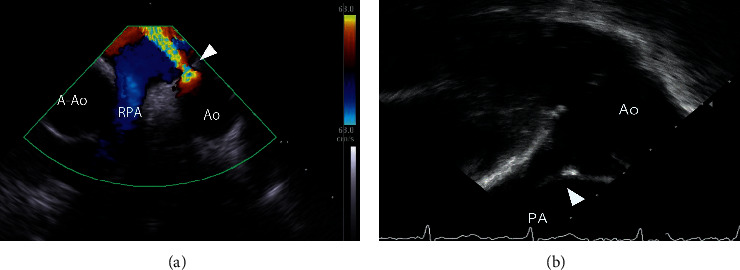
Representative image of MPA and LPA views of PDA using ICE. (a) The MPA view shows the aortic short axis view. (b) The LPA view shows the PDA long axis. A‐Ao, ascending aorta; Ao, aorta; ICE, intracardiac echocardiography; LPA, left pulmonary artery; MPA, main pulmonary artery; PA, pulmonary artery; PDA, patent ductus arteriosus.

The MPA view showed the aortic short axis, including the PDA, orifice of the LPA and right pulmonary artery, and descending aorta (Figure 1(a)) [16]. Further, we advanced ICE catheter into LPA to obtain the LPA view that showed the PDA long axis because the LPA runs parallel to the aorta (Figure 1(b)).

**Figure 2:**
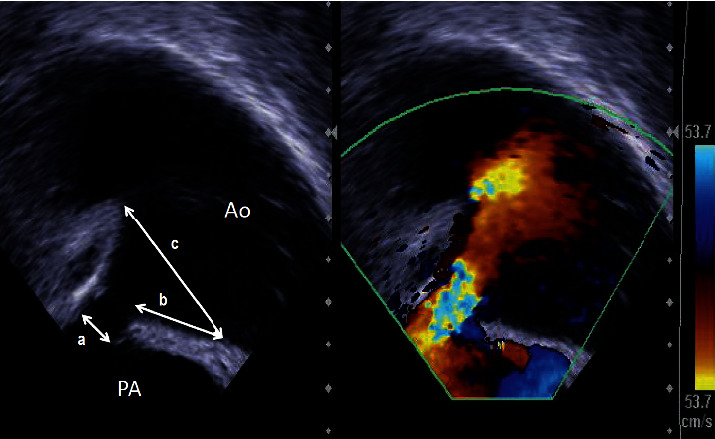
PDA measurement methods in LPA view using ICE. (A) The pulmonary artery side diameter of the PDA. (B) The lower contour PDA length between the end of the PA and the starting point of the aortic ampulla. (C) The aortic side diameter of the PDA. Ao, aorta; ICE, intracardiac echocardiography; PA, pulmonary artery; PDA, patent ductus arteriosus; LPA, left pulmonary artery.

We measured the PA side diameter, length, and aortic side diameter of the PDA using the MPA or LPA view (Figure 2) in all patients. In this study, length was defined as the lesser curvature length of the PDA, between its pulmonary and aortic ends. We measured the maximum values of these during systole.

Based on the ICE measurements, we selected the device, its size, and the approach. When Amplatzer occluders (AGA Medical, Golden Valley, MN, USA) were selected, their TC-PDA was performed in an antegrade fashion. While ICEs show LPA views, we advanced another 5 Fr multipurpose catheter (MP 3.0, Medikit, Miyazaki, Japan) from the femoral vein into the PDA, with the aid of a snare-wire from the aorta in many of the adult cases [19] and then replaced it with a long sheath. When a coil was selected, TC-PDA was performed on the patient in a retrograde fashion. A multipurpose catheter was retrogradely placed from the aorta into the PDA.

With ICE monitoring, we deployed the device into the PDA. Once the device was in a good position, we released it if there was no or minimal residual shunting. After 5 to 10 min, we checked the residual shunt using ICE and completed the TC-PDA. We recorded the outcome of the procedure and any complications, including arrhythmias, cardiac or vascular injuries, and cardiac effusions.

### 2.4. Phase 1

We compared measurements between ICEs and aortographies or CCTs in PA side diameter, PDA length, and aortic side diameter. Based on a previous study [16], we presumed a very high correlation coefficient in PA side diameter between ICE and aortographies or CCT, but 0.5 to 0.6 of correlation coefficient in Ao side diameter. Therefore, the sample size of this study was determined as at least 19 patients with 0.05 of *α* error rate and 0.20 of *β* error rate [20].

In aortography, the PA side diameter, length, and aortic side diameter were measured during systole when the PA side of the PDA achieved the largest diameter in a given cardiac cycle. In CCT that was offered before TC-PDAs in our institutions or the other institutions, PDA sizes were measured in the long axis view of the PDA.

To determine the intra- and interobserver variability for ICEs, 10 patients were randomly selected, and the measurements were taken by 2 separate occasions and by 2 observers (H. Y. and Y. M.) who were blinded to the aortography or CCT results.

### 2.5. Phase 2

In phase 2 of this study, we compared the efficacy and safety of TC-PDAs performed with ICE guidance (*N* = 10) and those performed without ICE guidance (*N* = 16). We compared the age, body weight, PDA size, pulmonary to systemic blood flow ratio, mean pulmonary artery pressure, pulmonary artery resistance, fluoroscopic time, contrast volume, comorbidities, medications, and any complications between the 2 groups.

### 2.6. Statistical Analysis

Data were presented as medians with ranges or means ± standard deviations, as appropriate. For phase 1, we determined Pearson's correlation coefficient for PDA sizes between the ICEs and aortographies or CCTs. We used a Bland–Altman plot and Wilcoxon test to analyze the measurement agreements between the imaging modalities. To determine intra- and interobserver variabilities, we determined intra- and interclass correlation coefficients. The Mann–Whitney rank-sum test was used in phase 2 of this study. Statistical significance was considered as *p* < 0.05. Data analyses were performed using JMP 14.0 (SAS Institute, Cary, NC, USA) and IBM SPSS Statistics, Ver. 20.0 (Japan IBM, Tokyo, Japan).

## 3. Results

Phase 1 patient demographics are shown in Table 1. The median age was 17 years (range: 4.6–85 years), median Qp/Qs (Qp: pulmonary flow, Qs: systemic flow) was 1.6 (1.1–2.42), and median pulmonary artery pressure was 17 mmHg (range: 11–64 mmHg). Based on aortography or CCT, the PDA shape was classified as Krichenko type A in 19 (83%) patients, type B in 1 (patient 23), type *D* in 1 (patient 14), and type *E* in 2 patients (patients 1 and 9) [21].

### 3.1. ICE-Guided TC-PDA

All patients underwent successful TC-PDAs based on the ICE measurements. The ICE catheter we mainly used was the Acu Nav™ (Biosense Webster Inc., Diamond Bar, CA, USA) except for the ViewFlexXtra (St. Jude Medical, St. Paul, MN, USA) in one patient. We placed an Amplatzer duct occluder (ADO, AGA Medical, Golden Valley, MN, USA) in 21 patients (Patients 2 to 22) with a PA side diameter ≥2 mm and a Flipper coil (FC, Cook cardiology, IN, USA) in one patient (patient 1) with a PA side diameter <2 mm.

**Figure 3:**
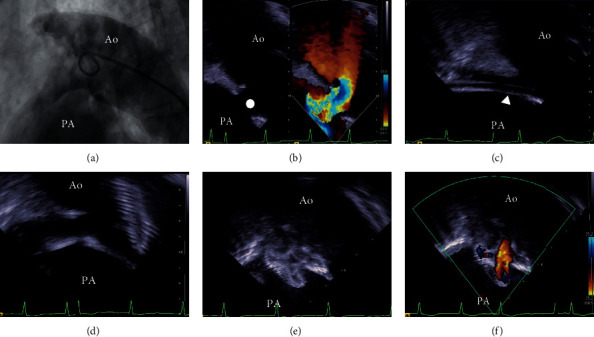
Representative case where ICE is a valuable guide for TC-PDA. Patient 23 has a large Krichenko type B PDA that was not delineated by an aortography (a) but was clearly visualized by ICE (closed circle) with a 11.1 mm PA side diameter and a 3.9 mm length (b). We chose Amplatzer septal occluder 17 mm for the closure of this PDA. During TC-PDA, ICE showed all process of procedure. We advanced a long sheath (arrowhead) through the PDA (c), opened the aortic skirt in the aorta (d), and pulled the entire system back and deployed right atrial disc (e). Finally, ICE showed small residual shunt just beside the body of device (f) and we finished the procedure successfully. Ao, aorta; ICE, intracardiac echocardiography; PA, pulmonary artery; PDA, patent ductus arteriosus; TC- PDA, transcatheter closure of patent ductus arteriosus.

In Patient 23, who had a large PDA and pulmonary hypertension, with a mean pulmonary artery pressure of 64 mmHg and pulmonary vascular resistance of 7.6 Wood UM [2], we placed an Amplatzer septal occluder (ASO, AGA Medical Corporation, Golden Valley, MN, USA). In this case, ICE was a valuable guide for TC-PDA. An aortography could not delineate the PDA well (Figure 3(a)), but ICE visualized Krichenko type B PDA with 11.1 mm of PA side diameter and 3.9 mm length (Figure 3(b)). Based on our experience, we chose the 17 mm Amplatzer septal occluder to minimize the risk of embolization of the device and avoid extensive protrusion of the left atrial disc into the aorta. During the TC-PDA, the ICE showed the whole process of the device placement. We passed a long sheath through the PDA (Figure 3(c)), opened a skirt in the aorta (Figure 3(d)), pulled back the entire system, and deployed the right atrial disc in the pulmonary artery (Figure 3(e)). Finally, ICE showed a small residual shunt close to the body of the device (Figure 3(f)), and we completed the procedure successfully.

Notably, 7 adult patients, including 1 with chronic kidney disease, underwent TC-PDAs with ICE guidance and without aortography. There were no major complications associated with ICE guidance such as arrhythmias, cardiac perforations, and vascular access problems that required specific treatments in this study.

### 3.2. Phase 1/Comparison of Measurements

The comparable measurements by ICEs and aortographies or CCTs are shown in Table 2. Of 23 patients, 14 patients with adult body size underwent CCTs before the TC-PDA in which the protocol was institution-dependent with various slice thicknesses ranging from 1.25 to 3.0 mm with a median of 2.0 mm. Of these 14 patients, measurements were taken by CCTs in 8 patients, patients 10, 11, 15, and 19–23, because the CCT images were appropriate for the measurements. In the remaining 15 patients, patients 1–9, 12–14, and 16–18, the measurements were taken using aortography.

**Figure 4:**
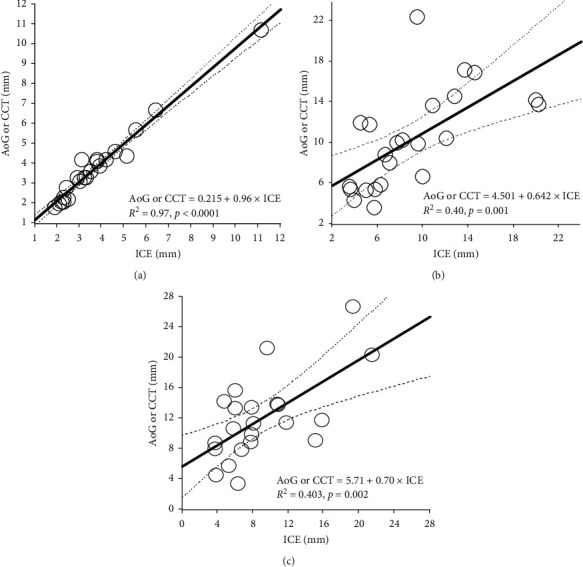
Correlations of measurements between the ICE and aortography or CCT. There was significantly highly positive correlation in measurements of the PA side diameter between ICEs and aortographies or CCTs ((a) *r* = 0.985, 95% confidence interval 0.967–0.993, *p* < 0.0001), but moderate correlation in measurements of the length ((b) *r* = 0.653, 95% confidence interval 0.356–0.830, *p* < 0.0003) and aortic side diameter of the PDA ((c) *r* = 0.704, 95% confidence interval 0.435–0.858, *p* < 0.0001) between them. AoG, aortography; CCT, cardiac computed tomography; ICE, intracardiac echocardiography; PA, pulmonary artery; PDA, patent ductus arteriosus.

The correlations between these measurements are shown in Figures 4(a)–4(c). We found significantly highly positive correlation in measurements of the PA side diameter between ICEs and aortographies or CCTs (Figure 4(a), *r* = 0.985, 95% confidence interval 0.967–0.993, *p* < 0.0001), but moderate correlation in measurements of the length (Figure 4(b), *r* = 0.653, 95% confidence interval 0.356–0.830, *p* < 0.0003) and aortic side diameter of the PDA (Figure 4(c), *r* = 0.704, 95% confidence interval 0.435–0.858, *p* < 0.0001) between them.

**Figure 5:**
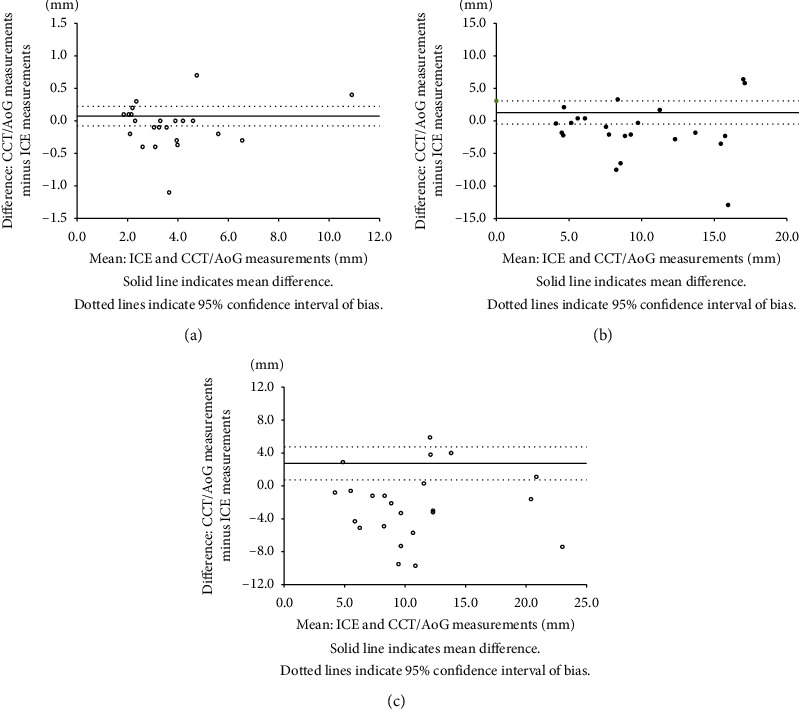
Bland–Altman plots between the ICE and aortography or CCT. On the Bland–Altman plot, there were the greatest agreement in the PA side diameter between ICE and aortography or CCT ((a) mean bias 0.074 mm with 95% confidence interval −0.077 to 0.224), but moderate agreement in length ((b) mean bias 1.287 mm with 95% confidence interval −0.491 to 3.065) and poor agreement in the aortic side diameter ((c) mean bias 2.735 with 95% confidence interval 0.738 to 4.732) with statistically significantly higher value measured by aortography or CCTs (*p* < 0.01). AoG, aortography; CCT, cardiac computed tomography; ICE, intracardiac echocardiography; PA, pulmonary artery; PDA, patent ductus arteriosus.

Using the Bland–Altman plot, we found great agreement in the PA side diameter between ICE and aortography or CCT (Figure 5(a), mean bias 0.074 mm with 95% confidence interval -0.077 to 0.224), but moderate agreement in length (Figure 5(b), mean bias 1.287 mm with 95% confidence interval 0.491 to 3.065) and poor agreement in the aortic side diameter (Figure 5(c), mean bias 2.735 with 95% confidence interval 0.738 to 4.732), with statistically significantly higher values measured by aortography or CCTs (*p* < 0.01).

The intraclass correlation coefficients were 0.989 (95% confidence interval 0.959–0.997, *p* < 0.000) for PA side diameter, 0.846 (95% CI 0.521–0.959, *p* < 0.000) for PDA length, and 0.887 (95% CI 0.630–0.970, *p* < 0.000) for aortic side diameter, indicating acceptably high intraobserver agreements. On the other hand, the interclass correlation coefficients were 0.992 (95% CI 0.967–0.998, *p* < 0.000) for PA side diameter, 0.663 (95% CI 0.102–0.904, *p*=0.01) for PDA length, and 0.337 (95% CI −0.332–0.781, *p*=0.15) for aortic side diameter, indicating poor interobserver agreements for aortic side diameter.

### 3.3. Phase 2

In phase 2 of this study, TC-PDA required significantly lower contrast volume when it was ICE-guided than when it was not because 7 patients with ICE guidance underwent TC-PDA without any contrast angiography, and there was no significant difference in demographics including age, PDA size, pulmonary to systemic blood flow ratio, and fluoroscopic time between the 2 groups (Table 3).

Of note, in 2 patients of TC-PDA without ICE guidance, the operator inadvertently opened the body of ADO in the aortic ampulla under fluoroscopy and that was recognized by pulmonary arteriography to confirm the position of device. Then, the device was further pulled into ampulla so that body of ADO protruded enough in the pulmonary artery. This type of problem could be easily appreciated by TC-PDA with ICE guidance because we could continuously monitor the device position in relation to the surrounding vascular structures.

There were no major complications during or after the procedure of TC-PDA with ICE guidance; however, in the TC-PDA without ICE guidance group, we encountered a complication of iliac artery dissection that improved with time. No other major complications were observed in both groups.

A patient in the TC-PDA with ICE guidance group developed a minor complication in which there was large groin hematoma that resolved spontaneously with time. On the other hand, in the TC-PDA without ICE guidance group, there were 4 complications recorded during or after the procedure. These included a large groin hematoma that resolved spontaneously, a postprocedure hypertension that required an intravenous calcium channel blocker, a transient supraventricular tachycardia that resolved spontaneously, and a transient hemolytic anemia that resolved spontaneously.

## 4. Discussion

This study indicated that ICE provided accurate PDA measurements in PA side diameter, which were comparable with aortography or CCT procedures. ICE can be a safe alternative to conventional aortography as a TC-PDA guide.

In phase 1 of this study, we found great correlation and agreement between ICE and aortography or CCT for PA side diameter measurements, but moderate correlation and agreement in length and moderate correlation and poor agreement in Ao side diameter.

Nevertheless, the ICE images produced in this study were used as practical guides for selecting devices for TC-PDA. Generally, we selected the device type and size based on the PA side diameter and length of the PDA, which showed satisfactory agreement between ICE and aortography or CCT measurements. Therefore, we could have used ICE to select the appropriate devices and successfully closed all PDAs in this study. In contrast, the aortic side diameter may not be an important factor in selecting devices in adult patients, because the aortas are generally dilated and can accommodate large devices without aortic blood flow obstruction, especially for Krichenko type A and type B PDA.

The differences between the PDA sizes measured by ICE and aortography or CCT are influenced by several factors. For ICE measurements, the distance between the ICE probe and the measured body structure affects by the accuracy of the measurements: the closer the distance, the more accurate the measurements, which is a characteristic of echocardiography. Therefore, the moderate correlation and poor agreement between the aortic side diameters measured by ICE and aortography or CCT is reasonable. On the other hand, the aortic side diameters of PDAs on aortography may not be accurate because aortography uses a projection image. The distance that we measured on aortography may not be accurate on a single cross section. Therefore, CCT should provide more accurate PDA measurements. However, the protocols for image acquisition of CCTs were not uniform in this study. The image thickness ranged from 1.25 to 3.0 mm, and the image acquisition timing was not the same. These variabilities might have further increased the differences in the aortic side diameters between the ICEs and CCTs.

Nevertheless, as shown in Figure 3, we confirmed that ICE is a valuable guide for TC-PDAs. In this case, aortography could not delineate the PDA, but ICE visualized its anatomy. In addition, ICE permitted the appreciation of the spatial relationship between the device and the PDA during TC-PDA [16]. Conventional fluoroscopic guidance is associated with a degree of uncertainty because we have to presume the position of the PA side of the PDA in relation to the tracheal shadow and rely on our intuitions as we open the body of the occluder. ICE eliminates these uncertainties because it provides comprehensive monitoring.

Consequently, the advantages of the ICE guide for TC-PDA are multifaceted. First, ICE guidance allows interventionists to place the device conveniently and accurately because it directly visualizes the anatomy of the PDA and the spatial relationship between the device and PDA. Therefore, ICE guidance should be ideal for large PDAs that cannot be delineated by aortography and less experienced interventionists. Second, ICE guidance eliminates the risk of contrast-related adverse events such as nephropathy and anaphylaxis and is preferred for patients with chronic kidney disease, occasionally seen in aged patients and patients with a significant history of allergy. Finally, by observing the vascular wall of the pulmonary artery, PDA, and aorta, ICE may facilitate the detection of vascular complications such as aortic dissections during TC-PDA [22, 23]; this is not possible with fluoroscopy.

The disadvantages of ICE guidance include limited echo views, invasiveness, and cost. Although ICE was useful for measuring the size of PDAs in this study, it may be difficult to visualize the entire PDA in different orientations. The use of ICE during the procedure requires additional venous access for the catheter introduction, and it may require time and practice to obtain adequate images. In addition, advancing the ICE catheter into the pulmonary artery via the right ventricle might increase risks, including cardiovascular injuries and arrhythmias. Careful ICE catheter manipulation is mandatory. Advancing the ICE catheter through a preplaced long sheath in the pulmonary artery should reduce these risks, although major complications did not occur in this study. While the ICE catheter adds cost to the procedure, reducing the number of contrast aortographies and procedure durations may be beneficial. Further accumulation of data and investigations are required.

### 4.1. Limitations

There were several limitations to this study. First, reference imaging modalities were either aortographies or CCTs and these were not the same, although both are standard modalities for measuring PDA sizes. Aortography could not visualize the contours of large PDAs in adults; therefore, it was not offered to all patients. Conversely, we knew that aortography was usually enough to visualize PDAs in pediatric patients; thus, we did not offer CCTs to those patients to avoid unnecessary irradiation. Secondly, as previously indicated, the institutional CCT acquisition protocols were not uniform. Practically, it was difficult to ask all institutions to follow the same CCT protocol because of their internal institutional policies. Finally, in this study, nearly all patients had Krichenko type A PDAs. However, even in patients with types B, D, and E, ICE provided reasonable measurements and was a valuable guide for TC-PDAs, although the numbers were small.

## 5. Conclusions

ICE was comparable to aortography or CCT in providing accurate measurements of PA side diameter for TC-PDA. ICE may be a safe alternative to conventional imaging as a guide for TC-PDAs and can be useful for patients with large PDA, renal dysfunction, or contrast allergy.

## Figures and Tables

**Table 1:** Demographics of the subjects.

#	PA side diameter								
	Age	Body height	Body weight	Measured by ICE			Mean PAP	Rp	Devices
	(y. o.)	(cm)	(kg)	Sex	(mm)	Qp/Qs	(mmHg)	Wood · U	
1	17	159	52.8	F	1.9^M^	1.4	18	1	Coil
2	8.9	134	34	F	2,0^M^	1.2	16	1.5	ADO (6/4)
3	8.4	119.3	20.3	F	2.1^L^	1.6	16	1.5	ADO (6/4)
4	9.5	136.5	27.1	F	2.2^L^	1.4	24	3	ADO (8/6)
5	5.7	98.7	15.9	F	2.3^L^	1.2	16	1.2	ADO (5/4)
6	5.1	111	19.7	F	2.3^L^	1.1	26	1.8	ADO (5/4)
7	5.3	111.7	18.7	M	2.4^L^	1.4	17	1.2	ADO (6/4)
8	6.2	120	25.3	M	2.5^M^	1.3	17	1.7	ADO (6/4)
9	4.6	106	17.8	F	2.9^M^	1.7	16	1.9	ADO (6/4)
10	14.7	157.3	49.8	F	3.0^L^	1.1	14	1.5	ADO (8/6)
11	39.1	166.4	73.2	M	3.1^L^	1.4	19	2.3	ADO (10/8)
12	8.9	112	18.3	M	3.2^L^	1.7	29	2.9	ADO (8/6)
13	17.9	156	42.3	F	3.3^L^	1.2	22	1.9	ADO (10/8)
14	48.5	169	58.6	M	3.5^M^	1.7	11	0.4	ADO (8/6)
15	35.4	173	65.9	M	3.8^L^	2.4	29	2.5	ADO (8/6)
16	76.5	151	46.5	M	3.8^M^	2.2	18	2.1	ADO (10/8)
17	13.2	143	34	M	3.9^M^	1.8	17	0.9	ADO (8/6)
18	85	144	46.2	F	4.2^L^	2.1	27	2.2	ADO (12/10)
19	63.7	160	56.0	F	4.6^L^	1.9	14	2.3	ADO (10/8)
20	57.4	149.6	58.5	F	5.1^L^	1.4	17	2.7	ADO (10/8)
21	31.1	170.6	51.7	M	5.5^L^	1.7	15	1.2	ADO (10/8)
22	66.1	153	41.7	F	6.4^L^	2.2	17	3	ADO (12/10)
23	62	145	31.2	F	11.1^L^	1.8	64	7.1	ASO (17)

The shape of PDA was classified as Krichenko type A in most patients except for type B in Patient 23, type *D* in patient 14, and type *E* in patient 1 and 9. ^L^Measured by left pulmonary artery view. ^M^ Measured by main pulmonary artery view. ADO, Amplatzer duct occluder; AoG, aortography; ASO, Amplatzer septal occluder; CCT, cardiac computed tomography; F, female; M, male; PAP, pulmonary artery pressure; PDA, patent ductus arteriosus; Rp, pulmonary artery resistance; *y*. o., years old; #, patient number.

**Table 2:** ICE versus aortography or CCT in measuring PDA size.

#	Pulmonary side diameter (mm)		PDA length (mm)		Aortic side diameter (mm)	
	ICE	AoG/CCT	ICE	AoG/CCT	ICE	AoG/CCT
1	1.9^M^	1.8^A^	8.2^M^	10.3^A^	3.8^M^	4.6^A^
2	2,0^M^	2.2^A^	10^M^	6.7^A^	15.0^M^	9.1^A^
3	2.1^L^	2.0^A^	5.7^L^	3.6^A^	6.3^L^	3.4^A^
4	2.2^L^	2.1^A^	6.7^L^	8.8^A^	3.7^L^	8.8^A^
5	2.3^L^	2.1^A^	3.5^L^	5.7^A^	5.2^L^	5.8^A^
6	2.3^L^	2.3^A^	5.0^L^	5.3^A^	3.7^L^	8.0^A^
7	2.4^L^	2.8^A^	4.5^L^	12.0^A^	6.0^L^	13.3^A^
8	2.5^M^	2.2^A^	5.8^M^	5.4^A^	14.0^M^	10.2^A^
9	2.9^M^	3.3^A^	7.1^M^	8.0^A^	10.7^M^	13.9^A^
10	3.0^L^	3.1^C^	20.2^L^	13.8^C^	9.6^L^	21.2^C^
11	3.1^L^	4.2^C^	5.3^L^	11.8^C^	4.7^L^	14.2^C^
12	3.2^L^	3.3^A^	6.3^L^	5.9^A^	7.8^L^	9.9^A^
13	3.3^L^	3.3^A^	12.1^L^	10.4^A^	10.8^L^	13.8^A^
14	3.5^M^	3.6^A^	14.6^M^	16.9^A^	6.0^M^	15.7^A^
15	3.8^L^	4.1^C^	9.5^L^	22.4^C^	7.8^L^	13.5^C^
16	3.8^M^	4.2^A^	10.9^M^	13.7^A^	8.0^M^	11.3^A^
17	3.9^M^	3.9^A^	3.6^M^	5.4^A^	5.8^M^	10.7^A^
18	4.2^L^	4.2^A^	20.0^L^	14.2^A^	15.8^L^	11.8^A^
19	4.6^L^	4.6^C^	12.8^L^	14.6^C^	7.7^L^	8.9^C^
20	5.1^L^	4.4^C^	9.6^L^	9.9^C^	6.7^L^	7.9^C^
21	5.5^L^	5.7^C^	13.7^L^	17.2^C^	21.4^L^	20.3^C^
22	6.4^L^	6.7^C^	7.7^L^	10.0^C^	19.3^L^	26.7^C^
23	11.1^L^	10.7^C^	3.9^L^	4.3^C^	11.7^L^	11.4^C^
Median	3.2	3.3	7.7	10	7.8	11.3
IQR	2.3–4.2	2.2–4.2	5.3–12.1	5.7–13.8	5.8–14.0	8.8–13.9

^A^Measured by aortography. ^C^Measured by cardiac computed tomography. ^L^Measured by left pulmonary artery view. ^M^Measured by main pulmonary artery view. AoG, aortography; CCT, cardiac computed tomography; ICE, intracardiac echocardiography; IQR, interquartile range; PDA, patent ductus arteriosus; #, patient number.

**Table 3:** Comparison between TC-PDA with and without ICE guidance.

	With ICE guidance *N* = 10	Without ICE guidance *N* = 16
Age (years old)	35.4–85.0 (62.8)	25.1–82.6 (64.1)
Sex (male/female)	5/5	5/11
Body weight (kg)	29.0–73.2 (50.7)	35.0–76.0 (50.7)
Size of PA side PDA (mm)	3.2–11.7 (4.5)	2.3–9.0 (3.75)
Pulmonary to systemic flow ratio	1.4–2.4 (1.9)	1.1–2.9 (1.8)
Mean PA pressure (mmHg)	11–64 (18.5)	10.0–34.0 (20.0)
PVR (U · M^2^)	0.7–7.0 (2.1)	0.8–4.2 (1.6)
Fluoroscopic time (minutes)	35.0–70.7 (49.7)	14.0–65.2 (39.0)
Contrast volume (ml/kg)	0–2.4 (0.0)^*∗*^	1.9–6.6 (3.0)
Comorbidities	PH 3, AF 2, CKD 2	Hypertension 2, AF 1
	Hypertension 2 s/p PDA 1, MR 1	PH 1, MR 1
	s/p TAPVR + res ASD 1	Basedow's disease 1
	Epilepsy 1	Hypothyroidism 1
		Congenital rubella synd. 1
		Congenital CMV infection 1
		Schizophrenia 1
Medications	DOAC 3	
	Antihypertensives 3	
	Diuretics 3	
	Anticonvulsant 1	
	Anti-PH drug 1	

^*∗*^ *p* < 0.0001 versus without ICE guidance. AF, atrial fibrillation; CKD, chronic kidney disease; CMV, cytomegalovirus; DOACs; non-vitamin K antagonist direct oral anticoagulants, ICE, intracardiac echocardiography; MR, mitral regurgitation; PA, pulmonary artery; PDA, patent ductus arteriosus; PH, pulmonary hypertension; PVR, pulmonary vascular resistance; TAPVR, total anomalous pulmonary venous return.

## Data Availability

The data used to support the findings of this study are included within the article.

## References

[1] Porstmann W., Wierny L., Warnke H. (1967). Closure of persistent ductus arteriosus without thoracotomy. *German Medical Monthly*.

[2] Baumgartner H., Bonhoeffer P., De Groot N. M. (2010). ESC Guidelines for the management of grown-up congenital heart disease (new version 2010). *European Heart Journal*.

[3] El-Said H. G., Bratincsak A., Foerster S. R. (2013). Safety of percutaneous patent ductus arteriosus closure: an unselected multicenter population experience. *Journal of the American Heart Association*.

[4] Farooqi M., Stickley J., Dhillon R. (2019). Trends in surgical and catheter interventions for isolated congenital shunt lesions in the UK and Ireland. *Heart*.

[5] Feltes T. F., Bacha E., Beekman R. H. (2011). Indications for cardiac catheterization and intervention in pediatric cardiac disease. *Circulation*.

[6] Moore J. W., Levi D. S., Moore S. D., Schneider D. J., Berdjis F. (2005). Interventional treatment of patent ductus arteriosus in 2004. *Catheterization and Cardiovascular Interventions*.

[7] Morgan-Hughes G. J., Marshall A. J., Roobottom C. (2003). Morphologic assessment of patent ductus arteriosus in adults using retrospectively ECG-gated multidetector CT. *American Journal of Roentgenology*.

[8] Shafi N. A., Singh G. D., Smith T. W., Rogers J. H. (2018). Sizing of patent ductus arteriosus in adults for transcatheter closure using the balloon pull-through technique. *Catheterization and Cardiovascular Interventions*.

[9] Galeczka M., Szkutnik M., Bialkowski J. (2020). Transcatheter closure of patent ductus arteriosus in elderly patients: initial and one-year follow-up results-do we have the proper device?. *Journal of Interventional Cardiology*.

[10] Goss J. E., Chambers C. E., Heupler F. A. (1995). Systemic anaphylactoid reactions to lodinated contrast media during cardiac catheterization procedures: guidelines for prevention, diagnosis, and treatment. *Catheterization and Cardiovascular Diagnosis*.

[11] Sardinha D. M., Simor A., de Oliveira Moura L. D. (2020). Risk factors for acute renal failure after cardiac catheterization most cited in the literature: an integrative review. *International Journal of Environmental Research and Public Health*.

[12] Glassman E., Kronzon I. (1981). Transvenous intracardiac echocardiography. *The American Journal of Cardiology*.

[13] Bartel T., Muller S., Biviano A., Hahn R. T. (2014). Why is intracardiac echocardiography helpful? Benefits, costs, and how to learn. *European Heart Journal*.

[14] Hijazi Z. M., Wang Z., Cao Q.-L., Koenig P., Waight D., Lang R. (2001). Transcatheter closure of atrial septal defects and patent foramen ovale under intracardiac echocardiographic guidance: feasibility and comparison with transesophageal echocardiography. *Catheterization and Cardiovascular Interventions*.

[15] Vaina S., Ligthart J., Vijayakumar M. (2006). Intracardiac echocardiography during interventional procedures. *EuroIntervention: Journal of EuroPCR in Collaboration with the Working Group on Interventional Cardiology of the European Society of Cardiology*.

[16] Kudo Y., Suda K., Yoshimoto H. (2015). Trans-pulmonary echocardiography as a guide for device closure of patent ductus arteriosus. *Catheterization and Cardiovascular Interventions*.

[17] Teramachi Y., Suda K., Yoshimoto H., Kishimoto S., Kudo Y., Iemura M. (2015). Transpulmonary echocardiography to guide stent implantation into coarctation of the aorta. *Echocardiography*.

[18] Yoshimoto H., Suda K., Kishimoto S., Kudo Y. (2016). Intra-cardiac echocardiography-guided stent implantation into stenosed superior vena cava in a patient with a history of contrast anaphylaxis. *Heart and Vessels*.

[19] Bentham J. R., Thomson J. D. R., Gibbs J. L. (2012). Transcatheter closure of persistent ductus arteriosus in adults. *Journal of Interventional Cardiology*.

[20] Hulley S. B. C. S., Browner W. S., Grady D., Newman T. B. (2013). *Designing clinical research: an epidemiologic approach*.

[21] Krichenko A., Benson L. N., Burrows P., Möes C. A. F., McLaughlin P., Freedom R. M. (1989). Angiographic classification of the isolated, persistently patent ductus arteriosus and implications for percutaneous catheter occlusion. *The American Journal of Cardiology*.

[22] Yamamoto H., Otake H., Akagi T. (2019). Acute aortic dissection as a rare complication of percutaneous closure using the amplatzer vascular plug II for a tubular and enlarged patent ductus arteriosus in an elderly patient. *JACC: Cardiovascular Interventions*.

[23] Yamamoto H., Shinke T., Otake H., Tanaka H., Matsumoto K., Hirata K.-i. (2019). Acute ascending aortic dissection due to transcatheter patent ductus arteriosus closure in the elderly: an extremely rare complication of transcatheter patent ductus arteriosus closure. *Journal of Cardiology Cases*.

